# Clinical researchers’ lived experiences with data quality monitoring in clinical trials: a qualitative study

**DOI:** 10.1186/s12874-021-01385-9

**Published:** 2021-09-20

**Authors:** Lauren Houston, Ping Yu, Allison Martin, Yasmine Probst

**Affiliations:** 1 School of Medicine, Faculty of Science, Medicine and Health, University of Wollongong, Northfields Ave, Wollongong, NSW 2522 Australia; 2 Illawarra Health and Medical Research Institute, University of Wollongong, Northfields Ave, Wollongong, NSW 2522 Australia; 3 School of Computing and Information Technology, Faculty of Engineering and Information Science, University of Wollongong, Northfields Ave, Wollongong, NSW 2522 Australia

**Keywords:** Clinical research, Clinical study, Clinical trial, Observational study, Data quality, Information quality, Good clinical practice, Education and training, Data monitoring, Data management

## Abstract

**Background:**

Fundamental to the success of clinical research that involves human participants is the quality of the data that is generated. To ensure data quality, clinical trials must comply with the Good Clinical Practice guideline which recommends data monitoring. To date, the guideline is broad, requires technology for enforcement, follows strict industry standards, mostly designed for drug-registration trials and based on informal consensus. It is also unknown what challenges clinical trials and researchers face in implementing data monitoring procedures. Thus, this study aimed to describe researcher experiences with data quality monitoring in clinical trials.

**Methods:**

We conducted semi-structured telephone interviews following a guided-phenomenological approach. Participants were recruited from the Australian and New Zealand Clinical Trials Registry and were researchers affiliated with a listed clinical study. Each transcript was analysed with inductive thematic analysis before thematic categorisation of themes from all transcripts. Primary, secondary and subthemes were categorised according to the emerging relationships.

**Results:**

Data saturation were reached after interviewing seven participants. Five primary themes, two secondary themes and 21 subthemes in relation to data quality monitoring emerged from the data. The five primary themes included: education and training, ways of working, working with technology, working with data, and working within regulatory requirements. The primary theme ‘education and training’ influenced the other four primary themes. While ‘working with technology’ influenced the ‘way of working’. All other themes had reciprocal relationships. There was no relationship reported between ‘working within regulatory requirements’ and ‘working with technology’. The researchers experienced challenges in meeting regulatory requirements, using technology and fostering working relationships for data quality monitoring.

**Conclusion:**

Clinical trials implemented a variety of data quality monitoring procedures tailored to their situation and study context. Standardised frameworks that are accessible to all types of clinical trials are needed with an emphasis on education and training.

**Supplementary Information:**

The online version contains supplementary material available at 10.1186/s12874-021-01385-9.

## Background

Clinical trials involving human participants are crucial to the discovery of new health and disease outcomes [1]. Collecting high quality data is critical for the success of these studies. To verify that data is of a high quality, guidance is provided to clinical trials from the International Council for Harmonisation (ICH) Good Clinical Practice (GCP) guideline [2, 3]. The GCP guideline is the international, ethical and scientific standard for designing, conducting, recording and reporting trials that involve human participants. To ensure trials comply with the GCP guideline data monitoring is recommended. However, some studies have suggested that the GCP guideline is too broad, written to follow the strict industry standards, predominantly for drug-registration trials and grounded on an informal consensus rather than scientific evidence [4, 5]. Therefore, the resulting guideline is not suitable for certain types and contexts of clinical studies, such as non-drug intervention trials and observational studies.

Regardless of the study type or context, the 1996 GCP guideline recommended data monitoring should be performed on-site and using the method of source data verification (SDV) [3]. SDV requires the study staff to manually verify data points [6]. The method has been questioned due to on-site SDV being costly, time consuming in nature and failing to guarantee participant safety and data quality [7, 8]. Therefore, a new risk-based monitoring approach was promoted by the European Medicines Agency and the United States Food and Drug Administration in 2013 [9, 10]. An updated 2016 GCP E6(R2) guideline [2] now encourages clinical trials to incorporate a risk-based monitoring approach which is underpinned by information technology (IT) [11]. The application of IT in clinical studies has seen the emergence of a suite of data checking and aggregation packages [12] that has transformed data monitoring approaches. IT has allowed for real time data checking, quicker identification of missing data and statistical monitoring [13, 14]. However, it is largely unknown what challenges clinical studies and researchers face when implementing data monitoring approaches using IT systems.

Due to study complexity and regulatory scrutiny, it is increasingly difficult for clinical trials to monitor data quality. Prerequisite for efficient and high quality clinical research is knowledgeable and experienced researchers regardless of the study setting [15]. A joint task force has identified the core competency domains of clinical research, including study and site management, leadership and professionalism, and communication and teamwork [16]. This is in line with a risk-based monitoring approach which requires efficient teamwork, staff engagement and workflow to identify and resolve issues. However, within clinical study teams there may be miscommunication and duplication of effort due to the tendency to work in silos [17]. What is not yet clear is the impact of the clinical research environment on the data quality and study findings. This indicates a need to understand clinical researcher experiences with the working environment, the working procedures and their subsequent impact on data quality.

To the best of our effort, we only identified four qualitative studies that are focused on data monitoring procedures in clinical study settings [18–21]. Two of these studies have solely focused on the newly recommended risk-based approach; however, both studies questioned the cost-effectiveness of risk-based monitoring with concerns that infrequent on-site monitoring could miss queries of systematic error [18, 19]. Whilst the updated GCP guideline recommend risk-based monitoring, it is fair to say that there is a limited understanding on how clinical researcher experiences in implementing and working with such approaches, and their impact on data quality, warranting further investigation. In the Australian setting, quantitative data collected from cross-sectional surveys has found that small, single-site academic clinical trials implemented various non-standardised ad-hoc data monitoring procedures [22–24]. This current study was necessary to further explore the quantitative survey results and is the first to collect qualitative data from Australian clinical researchers about their experiences with data monitoring and meeting regulatory requirements. Thus, this study aimed to describe Australian researcher experiences with data quality monitoring in clinical trials. Herewith ‘data quality monitoring’ was defined as the oversight and review of research processes, procedures, records, data reporting, appropriate conduct and ongoing evaluation.

## Methods

### Study design

A mixed methods, explanatory sequential research design was employed [25]. This article presents the findings from the qualitative interviews. The quantitative survey results have been reported in the preceding article [23]. The decision to report the results separately was due to the timing of the sequential design and the findings being more clearly conveyed when presented separately. This approach was considered to be ideal to firstly, gain a general understanding of what data quality monitoring procedures were used in Australian clinical studies and secondly, to help explore the participant experiences, and elaborate on the quantitative findings. In the context of clinical practice, a study by Shneerson and Gale [26] reported that an explanatory sequential design allows researchers to refine qualitative research questions, explore the reasons for quantitative answers and ensure that the findings were meaningful. This mixed method approach also facilitates cross data validation.

The semi-structured interviews described in this study followed a guided-phenomenological approach which was deemed appropriate to explore clinical researcher commonalities as well as the structure and essence of the participant’s ‘lived experiences’ associated with data quality monitoring [27, 28]. Participants were considered experts and any new topics raised were explored in depth within the corresponding interview. The reporting of this study followed the COnsolidated criteria for REporting Qualitative research (COREQ) guidelines and checklist (Additional file 1) [29].

A semi-structured interview guide was developed with reference to the initial survey to collect open-ended data that was informed by the quantitative results reported by each survey respondent. The interview guide began with general questions asking the participants to describe their work experiences with clinical research. This was followed by their experiences with data quality monitoring (a) before the commencement of a clinical study, (b) during the data collection phase, (c) the methods applied, (d) during the data analysis phase and translation of data into information and (e) training and education received (Additional file 2). Probing questions were used to seek clarification. The interview guide was assessed for face-validity by a senior researcher (YP) prior to use. The interview guide was pilot tested via telephone with two colleagues (AM and SD) who worked with clinical trial research and had experience with data monitoring.

### Participant recruitment

An opportunity sample of Australian clinical researchers who had completed the initial quantitative survey [23] were invited to participate in the interviews. In brief, Australian clinical researchers listed on the Australian and New Zealand Clinical Trial Registry (ANZCTR) as the contact person for clinical study scientific queries [30] were contacted. Researchers listed were associated to clinical studies that met the following ANZCTR database eligibility criteria: all intervention (randomised and non-randomised) and observation trials; recruitment status ‘recruiting’ or ‘active, not recruiting’; all genders; all age groups; ethics approved; healthy and non-healthy volunteers; and the recruitment country of Australia. After completing the quantitative survey, the respondents were invited to participate in the interview. No previous relationship was established with participants prior to recruitment. It is recommended that phenomenological studies should interview five to 25 individuals who have experienced a phenomenon [31]. Therefore, all clinical researchers who expressed an interest were sent an invitation to participate and an outline of the interview questions (Additional file 3). Non-respondents were sent one single email reminder as follow up.

### Data collection

Telephone interviews were conducted between September 5, 2018 and October 22, 2018. Each interview was scheduled for a period of 30 to 60 min and no repeated interviews were conducted. Researcher (LH) had training in research theory and prior experience in observing qualitative research, thus conducted the interviews. As this study was part of LH’s doctoral research, she was familiar with the previous survey results and therefore employed the strategy of “bracketing” [27, 32] to set aside her own presumptions whilst remaining open to the reality experienced by the participants. To minimise bias LH wrote down her own views about data quality monitoring prior to proceeding with the interviews. During and immediately following each interview, ‘memos’ (or field notes) were documented to provide context (i.e. feelings, tone and ease of conversation) and preliminary thoughts about possible themes [33]. Ethics approval for the study was obtained from the University of Wollongong Human Research Ethics Committee (HE16/131). All participants provided written informed consent. Despite an offer made to interview participants to quality check their own transcripts, no participants elected for this option.

### Data analysis

All interviews were audio recorded and iteratively transcribed verbatim, removed of identifiers and checked for quality by an independent reviewer (CM, EM or DB) (see Additional file 4). All transcripts were uploaded, managed and reviewed using the qualitative analysis software QRS NVivo, version 11.0 (QSR International Pty Ltd., Doncaster, VIC, Australia). Inductive thematic analysis was employed to make sense of and build a narrative for the collected data [34]. Each transcript was analysed individually before the set of transcripts underwent thematic categorisation. This was to preserve the richness of each interviewee’s experience and to ensure that the analysis was grounded in the language of the participants. Themes were categorised as they became apparent, and memos were used to support the coding process. Thematic saturation was reached after seven participants were interviewed [35, 36]. Thematic saturation was determined at the point when themes were consistent across the varied perspectives and no newly added meaningful information was produced relative to the study objectives [37]. The primary data theme categorisation was coded by LH who discussed emergent themes with YP. Further, to check the robustness of the themes AM and PY independently reviewed and audited the themes for plausibility.

## Results

From the initial survey, 26 of the 441 (6%) survey respondents expressed an interest to participate in an interview. When contacted, four participants declined to participate, three due to time constraints and one did not give reason. A total of 15 participants did not respond to the email communication. Seven participants were interviewed, length of interviews ranging from 29 to 58 min (mean 42.1 ± 10.7 SD), and all were associated with intervention treatment clinical trials in an Australian setting (Table 1).

**Table 1:** Characteristics of participants and the associated clinical trial demographics

Participant					Clinical study						
Identifier	Gender	Highest education	Length of current employment (years)	Appointment	Organisation	Study type	Study phase^**a**^	Trial sites	Data collection setting(s)	International study^**b**^	Participants targeted for baseline enrolment
P1	Male	Doctoral	15	Continuing	H	I-T	4	Single	H	–	20–99
P2	Male	Doctoral	1	Fixed-term contract	A	I-T	4	Multi	H, PP	Yes	> 500
P3	Female	Postgraduate	8	Continuing	A	I-T	4	Multi	MS	No	100–499
P4	Female	Doctoral	6	Fixed-term contract	H	I-T	2	Multi	H, A	Yes	> 500
P5	Female	Doctoral	8	Fixed-term contract	A	I-T	3	Single	II	–	100–499
P6	Male	Doctoral	1.5	Fixed-term contract	A	I-T	NA	Multi	CS	No	> 500
P7	Female	Doctoral	2.5	Fixed-term contract	A	I-T	3	Single	H	–	100–499

Abbreviations: *A* academic (university); *CS* correction service; *H* hospital; *II* independent institute; *I-T* intervention (clinical) trial – treatment; *MS* medical service; *PP* private practice; *NA* not applicable

^a^ Study phase: Clinical trials of biomedical interventions typically proceed through four phases - 1, Phase I; 2, Phase II; 3, Phase III; 4, Phase IIII

^b^ Participants who selected ‘multi-site’ in the survey were asked if the study was part of an international study. Those who selected ‘single-site’ did not due to question branching logic

**Fig. 1:**
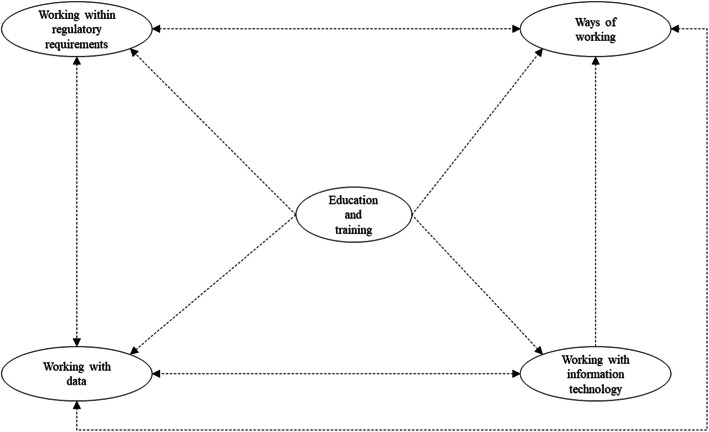
A thematic map of the relationships of the five primary themes

Five primary themes emerged from the interviews: (i) education and training, (ii) ways of working, (iii) working with IT, (iv) working with data and, (v) working within regulatory requirements. A thematic map (Fig. 1) was created to describe the relationships between the primary themes. Each primary theme is presented in an oval and broken lines reflect the relationship between the primary themes. The direction of the relationship is indicated by the arrow heads. The map illustrates the influence of ‘education and training’ on the other four primary themes. While ‘working with IT’ was seen to influence the ‘way of working’ theme, all other themes had reciprocal relationships. There was no relationship between ‘working within regulatory requirements’ and ‘working with IT’. The hierarchical structure of the primary themes (*n* = 5), secondary themes (*n* = 2) and subthemes (*n* = 21) are presented in Additional file 5. Herein forth the primary themes are shown in bold, secondary themes are bold and italicised, and the subthemes are italicised. Two secondary themes were created as higher order categories by grouping common and related subthemes. This followed a path to abstract the subthemes. A detailed list of the secondary themes, subthemes and representative quotes regarding each of the primary themes are shown in Tables 2 and 3.

**Table 2:** Primary themes, subthemes and representative quotes regarding the ‘Education and training’, “Ways of working’, ‘Working with technology’ and ‘Working within regulatory requirements’ primary themes

Primary theme	Subtheme	Exemplar quotes
Education and training	Importance of formal staff training	“Most organisations adhere to…GCP guidelines everybody is referring to the same bible [GCP] I suppose so you can’t stray too far from that” (P3)
		“I didn’t get any formal training. I suppose [I’m] just trying to be…I don’t know diligent and as careful as I could be conscientious with all the data in terms of ensuring that it would be [of] quality. I suppose and making sure we were sticking to ethics registration” (P5)
		“There isn’t a common basis for the whole lot. I think if you can get something there [education and training] and get people really thinking about it...I think people are yelling out for it” (P4).
		“If there’s more information or more training about data monitoring process or data entry education, to clinical research that would be very great.” (P6)
		“The only difference would of been…filling people in on the changes and then how that’s been incorporated into the standard operating procedures and their own unique way into each organisations.” (P2)
	Learning on the job	“So, you just develop over a period of time with that kind of exposure an understanding and appreciation of how these things [clinical study procedures] go.” (P2)
		“I think the most of my training really came about data quality monitoring really came with the audit. So, it wasn’t formal training, but it was a practical training.” (P7)
Ways of working	Responsibility	“we should come up as a team, make sure everybody knows their role that everything’s okay… make sure everything is in place” (P7)
		“I think you know if you’ve got clinicians so you know nurses putting in data they’re actually putting in data but they’re not really aware that data could be used in other things, and they don’t they don’t necessarily appreciate the importance of that all fields being completed.” (P4)
	Staff engagement	“That’s one very convenient way of engaging with a group of people who perhaps rarely get to play a role in helping put these things together…why don’t you review them and give them some comments” (P2).
		“We held regular meetings with staff so to ensure that there was any issue and if there were some yes if they’d obviously obtained an odd score or not response to a question, we could discuss it.” (P5)
		“I’ve talked about that with [boss] as I said even with the studies, I’m doing now I will go back to [boss] and say what do you think I should do with this? Um, how do you think I should manage this?” (P3)
	Organisational environment	“Whereas now I guess um I’ve moved up [laughs] it’s more the research assistants who are, who keep, keep an eye on it and I’m a little more distanced.” (P5)
	Skills and expertise	“People just aren’t intuitive with some things, it’s like you watch one person learn to drive and they’re terrible and others learn to drive and they’re a natural. Ultimately, people learn to drive but they’re at different paces. So, here we just worked out well some people are going to struggle with that instrument so we gave them as much information as we could. We did it visually [included pictures] because that’s much better than reading something so again we made it so it’s nice and quick.” (P1)
Working with technology	Technology induced changes	“I mean we used to in the old days, we would actually have to go to the sites to collate the papers that changed to then the papers would then be sent to us, so it started to get more about currency of data.” (P2)
		“So, it’s changed over time as I’m sure other participants would have well and truly told you. Um since the 90s when everything was paper based um you had…paper case report forms in duplicate.” (P3)
	Quicker and easier	“Yes, yes everything is there. So, we can just ah with because of everything is online everything from it is kept it is very easy to actually monitor.” (P7)
	Investment	“Real time range checking...it wouldn’t prohibit entry of data, but it would certainly require somebody to think about whether the number or the word they just put in was indeed the correct one.” (P2)
	Unintended consequences	“Make sure that…all the data had been entered correctly because at one stage you could enter it, but it wouldn’t go in…there was a glitch in it in the program.” (P4)
Working within regulatory requirements	Good clinical practice	“I made sure they actually did it [GCP training] although it was pretty tedious… it was like sticking pins in your eyes, but I actually still think it made people think about exactly what they’re doing and that they’re part of a bigger thing. I think if it was slightly tweaked, I think that, that it would be actually more instructive.” (P4)
		“It’s all been very repetitive, and it has all been very sensed around the same sort of rules…everything focuses back on GCP, so everybody keeps looking back to GCP and saying ok what are the requirements…what’s the bare minimal we can get away with.” (P3)
	Protocol	“I just follow the template…this was how it first did my, my first protocol” (P7).
		“That allowed us to adopt a whole range of more or less protocol defined approaches to all the activities relating to the design, implementation conduct and reporting of clinical trials.” (P2)
	Standard operating procedures	“You know and all it takes is…the irony…is you get on the internet, and you do a search for something like a standard operating procedure around a monitoring plan and you can get 10 or 20 different versions of the same thing on the internet and…you look at them with a fine-tooth comb and they all look very similar there’s not a lot of difference between them.” (P3)
		“It seems awfully difficult I don’t quite understand why we would want to do any or all of these things [SOPs]. Why can’t we just collect a truck load of data and then analyse it. So, um I think that’s an understandable thing um, but it requires a fair amount of work at the beginning. Particularly for new people.” (P2)
		“There was a commercial and non-commercial arm at the [location] and initially we had separate SOPs but then they all got moulded into one another. So, everything that use to be not quite as strict started to be become stricter and I think there was a lot of resentment around that actually in the team, including myself.” (P3)

**Table 3:** Themes, subthemes and representative quotes regarding the primary theme ‘Working with data’

Theme	Subtheme	Exemplar quotes
	Coping with data errors	“We’ve tried to minimise any bias, or you know introduction of any errors. So, we’ve always had the same training procedure” (P4).
		“We picked 1% probably arbitrarily…how much error would you begin to feel a bit uncomfortable about in terms of the capacity for seriously changing the reported outcome from a study” (P2).
		“Just because with excel there were quite a lot of ways things could go wrong like formulas that are set up in several spreadsheets or even jumping a line or just entering a wrong number. Just doing a typo which is not always visible straight away” (P5).
	Data audits	“We also, under various funding arrangements were subject to external completely independent compliance checks... we would welcome those and work very closely with the people doing it. We didn’t like them.” (P2)
	Coping with missing data	“I guess it makes it easier definitely the electronic way to see what’s missing…and I think it will save a lot of missing data.” (P5)
		“we would give them a call and ask over the phone, and usually we tried to do it within the um, within a two week period from the time we were supposed to have received it.” (P5)
		“I know that they did manage, that they managed to manipulate the data in such a way that they did get an outcome, but I know I remember we were struggling with that. I remember talking at meetings about how we were, how the statisticians were going to manage that to, to be able to provide an answer.” (P3)
Data monitoring	Monitoring approach	“doing some regular check, plotting the data, doing some simple stuff. So, descriptive stuff very regularly. Where I just got minimum maximum, you know approach and plotting the data to check it. Nothing was really um out of the ordinary.” (P5)
		“So, I have been involved in project they have they are very fussy about the data monitoring they have to check every day… probably back in the day its paper based…they didn’t check until the very end of the trial” (P6).
		“when we say monitoring, we are going to actually start implementing a lot more statistical compliance monitoring in house so we can save on travel because we are [name] funded so we don’t have a lot of funds to send people away.” (P3)
		“It depended on whether it was academic, whether it was commercial…investigator-initiated study, or an investigator sponsored study or a commercially sponsored study and what the aims of the study were” (P3).
		“I found that it varies from project to project and also ah even within the same setting ah you know different projects different research team um depending on their size may have different factors.” (P6)
		“Well, look um I am going to be sort of bold here and say, it’s never really has been different [data monitoring in the academic environment].” (P2)
		“I’ve always kinda taken the same approach in monitoring data quality” (P5).
		“We had to submit…a monitoring plan…this actually should have been submitted with the protocol, but we didn’t know at the time” (P7).
	Assumptions or opinions	“as opposed to a smaller um, experiments I guess where things, well things there’s no set date I guess and things can change at the start before you can do a lot of pilots I suppose before you start your ah your real data collection…there’s more freedom I suppose in changing things before you actually start” (P5).
		“They’re not a complicated study it’s not like a drug trial. Drug trials are the ones we have all those sorts of trouble” (P1).
Data quality	Elements of quality	“Unless you could substantiate claims about data integrity and reliability you really might as well not bother” (P2).
		“I remember even her [boss] saying ‘you know we don’t want to leave all this evidence around, sponsors to be looking at um and seeing that there’s of lots of dirty data sitting outside’ I don’t know if anyone else has said that to you but it’s something that has always stuck in my head, I always thought it was very interesting.” (P3)
	Factors influencing data quality	“the first thing you’d realise then is that CRFs would often lay around uncompleted for considerable periods of time and then there’d be a rush to fill them in before people arrived or they were due to be sent and inevitably when you allowed time to elapse between a clinical assessment and the forms being filled in there’s much greater chance of there being mistakes and errors.” (P2)
	Reporting data queries	“They [data collectors] are aware of the values they should be getting and what will be…outliers and they’re also I guess required to document everything [if] they think something strange happened during the visit and getting some odd values for an assessment. They take notes about what they think could be the cause for that at a later stage we can understand why this score will be an outlier.”(P5)

### Education and training

#### Importance of formal staff training

The importance of training and education arose from participant experiences of receiving training to meet regulatory standards. A few participants reflected on a lack of understanding of the importance of training and education, and suggested that more needs to be done. The following excerpt was echoed by the majority of participants:

“Everybody who does a clinical trial should have a basic training in you know GCP. You know it's a no brainer. It's sort of like you have you you're a dietitian or you're an exercise physiologist. Oh, and also this is your training [GCP] for this you know. That should be there” (P4).

There was consensus about the importance of staff training from organisation to organisation. Participants described that their training experience reflected the study context and the SOPs of their organisation in guiding them to complete tasks. However, one participant described:

“If you went from one organisation I’ve worked in, to another…the training would have been more or less the same” (P3).

#### Learning on the job

The analysis of participant responses suggested that clinical researchers did not receive formal training in data collection and data entry but instead learnt through absorption on the job. One participant portrayed this sentiment by stating:

“I'd say I picked it up on learning the trial itself. I picked it up on the job in terms of the data we were collecting and the methods” (P5).

### Ways of working

#### Responsibility

Participants stated the importance of providing staff ownership over their collected data as it was an opportunity for them to contribute to the study. This ownership would foster trust and create relationships between collaborating staff, sites or centres. Participants recognised that it was up to them and solely their responsibility:

“you're…never gonna get another chance to do this [clinical study] again. So, why not make sure that you do it right” (P2).

A few participants mentioned difficulties of working with clinicians whose research activities were an additional responsibility on top of their usual duties, which was seen to increase the clinicians’ workload. One participant confided:

“Once you have a clinician whose super imposing research for which they are not being paid and which they’re trying to squeeze into their usual day that’s when the issues arise” (P1).

#### Staff engagement

Several participants suggested that pilot testing was used as an approach to engage staff in the design and to allow them to familiarise with the study, which was described as useful and would positively impact study outcomes. However, one participant saw the effect of engaging with staff differently. It was a way to get to know and identify staff members:

“You know I was checking so I knew who was fudging stuff and who was actually doing it properly…you’d soon get them out of the way” (P4).

However, this same participant viewed the importance of teamwork, open conversations and feedback with their staff members as illustrated in their comment:

“So that always meant a discussion with all of us about how the workload was going to run and how we're going to handle the patient. So that was the most efficient use of our time and their time” (P4).

#### Organisational environment

Many participants felt that there was an organisational hierarchy in clinical research, which could create a disconnection between the levels of staff at the top and bottom. A participant experienced that senior staff members could lose touch with reality:

“I'm sort of in the middle you know of the tree…in reality only people who are doing the data capturing in the field are who know whether…the CRF [case report form] or the questionnaire there are actually feasible or not for the participants to fill in” (P6).

#### Skills and expertise

Involving skilled and expert staff members was ideal to interpret results of different tests. Participants described working in multidisciplinary studies which relied on the specialised staff members with the relevant training and education:

“People from several disciplines who were involved so there was a geriatrician who could interpret like the medication lists and suggest some recommendations. There was an exercise physiologist who could interpret and provide the recommendation with regard to the vestibular test performance often physiotherapist as well specialising in vestibular function or would be the one administering the vestibular rehabilitation and could give [their] opinion” (P5).

### Working with IT

#### Technology induced changes

There was widespread acknowledgement that the introduction of technology had changed the landscape of clinical studies. Participants advised that by adopting technology they had moved away from paper-based studies. This was described to be a positive experience as it upskilled staff, improved quality and reduced the number of checks:

“I really believe this…new system is going to help a lot… it’s reducing the checks, I think that are needed” (P5).

#### Quicker and easier

There was recognition that technology had encouraging effects as software could enable quicker and easier identification of data discrepancies and/or errors. There was also a benefit to having all data stored in a centralised system. One participant described:

“It's much easier to have quick look and know if there is much more [to be] checked on.” (P5).

#### Investment

Investing in technology allowed researchers to utilise functions including database locking, audit trails, preformatted fields and automatic range flags. Participants described functions as inevitably improving the efficiency of timely procedures including hand searching of paper documents.

“I think it depends, a bit on how you collect the data. For us because everything is collected in REDCap. So even for example, we have conditions and logic in place for when you put like height or weight [in]. So, it's something that if it's not between one meter and two meters, like two and a half meters it appears [as] a mistake like you cannot just put like a 100 [in]. It's going to appear as a mistake” (P7).

#### Unintended consequences

In some instances, instead of making improvement or bringing benefits the technology actually caused data loss. There was an understanding that different software systems were not compatible which adversely increased workloads. Feelings of frustration were raised related to how a software interface was designed. A participant described the hindrance caused by an unstable offline system:

“There's a chance that you might move the…data on the server and replace it with the empty data record on the iPad…that might lead to data loss on the server…which is a lot of trouble” (P6)*.*

### Working with data

#### Coping with data errors

Participants’ implemented different strategies to minimise error, which included measurement guidance, pictures on data collection forms, data ranges, real-time checking and sending all tests to a central place for analysis. However, accepting that humans make mistakes and that errors exist arose from the participants describing their experience with the process for data collection and transcribing data from paper into an electronic system. Technology was suggested to reduce human error although this relied on software configuration. In particular, a participant who utilised Excel spreadsheets for data storage expressed:

“I think if people are collecting [data on] paper and I'm not aware of what…researchers do that then…the margin for error is of course much much bigger because…you just need to accept there’s like a human mistake” (P7).

The expectations for clinical studies can factor into how researchers interpret the amount of error that is tolerable. One participant suggested that they selected an error acceptance level based on their individual opinion, while another participant suggested it was not possible to standardise an error acceptance level:

“I actually think that it [error acceptance level] depends on what therapeutic area you are working in, it depends on the risk of the intervention… it depends on the population you are testing in the intervention, it’s not just about the data, it’s situational. It really is a case-by-case basis…and I think to put a blanket rule down to say that this…level of error is acceptable is not possible. I think it just it has to really be assessed on a case-by-case basis” (P3).

#### Data audits

Participants who had been audited described this experience as unpleasant. Auditors were not liked, and the mandatory auditing process was considered to be scary. Although, one participant reflected on being audited as a positive learning experience and would recommend the procedure to other studies:

“it [being audited] was a very good process, I enjoy it. It was frightening like a bit scary, but I…learn a lot” (P7).

#### Coping with missing data

There was a general consensus that technology would aid in quicker identification of missing data by comparison to paper forms. However, no participants discussed calculating the amount of missing data before and after technology implementation. Furthermore, clinical researchers described strategies in place to overcome missing data. One strategy referred to by the participants was:

“[It was] predefined in the…monitoring plan as to how far back they [researchers/clinicians] can actually retrospectively ask patients for that data if it was missing. Ah, and if it could not be retrospectively collected, because it was outside the time allowed timeframe then it was identified as missing and the records actually stated that it was missing and there was no way of actually collecting that missing data”(P3).

Although it was vital to minimise missing data, it was also important to acknowledge that missing data does exist. A few participants described that they had no missing data points and everything was always complete. A sentiment echoed by one participant was illustrated as follows:

“We aren’t going to have any missing data points unless someone drops out, we aren’t going to have any missing data points. We just have a sheet you fill it out, you know they are all there if someone does miss let’s a say a subject for arguments sake haven’t put in or ticked a box on one of the questions then we would simply use the last value carried forward” (P1).

### Data monitoring

#### Monitoring approach

There was little consensus in data monitoring approaches among the participants, with some participants suggesting that the approach depended on the clinical situation and context. However, numerous participants described their approach was the same for different studies:

“There was no difference in the way the data were collected, how they were reviewed and the integrity was maintained. I just didn’t think there was any difference really” (P1).

Some participants expressed concerns that they had worked in organisations where no monitoring was undertaken. This was due to the monitoring not being seen as an important activity and a lack of knowledge on how to conduct data monitoring.

“I guess it's more in my head I suppose… I just knew what I needed to do. I never wrote it [monitoring procedure] down. I kind of just did the difference steps over and over” (P5).

The analysis also suggested that all participants had experience with ‘simple’ data checking. The frequency of data monitoring varied although the use of technology could lead to more frequent checking. Furthermore, the amount of monitoring was dictated by study funding, with one participant expressing that they had taken salary and staff cuts due to limited funding:

“We have run this study on the smell of an oily rag” (P4).

#### Assumptions or opinions

A few participants believed drug trials were more complicated. Additionally, commercial entities were suggested to be more stringent to the point that the amount of monitoring required was excessive. These participants had opinions that there was enough evidence to suggest that the amount of money, time and resources spent on certain monitoring methods was wasteful.

“I think in short commercial entities and working with commercial entities, the data monitoring activities have been a lot more stringent” (P3).

### Data quality

#### Elements of quality data

Participants felt a motivation and obligation to ensure that the data that was collected, stored and reported was legible and transparent. One participant described that they had witnessed staff ignorance about the data limitations and the criteria to judge a meaningful result:

“It just astounds me how ignorant people are of what the limitations are of their data and it's never discussed. I mean…with the waist measurement we always…measure from the bottom of the rib cage to the top of the hip and you take halfway…if you've got somebody who's obese…[and] you're doing it over the apron [this is a limitation]” (P4).

#### Factors influencing data quality

A few participants acknowledged that the goal of certain studies was not just about achieving good quality data but about forming a long-lasting relationship between services and participants. For example:

“[It’s] not just about data quality, ours [studies] are about creating a relationship with the services… and gaining trust in a community” (P3).

Additionally, the timeliness of data with the increased use of technology meant real time data collection allowed for improvements in quality and less missing data.

“The data is recorded straight away…we've got some questionnaires that are app based. So, I guess in this case you can't really influence the data, it’s really if a person is entering something wrong.” (P5)

#### Reporting data queries

Data queries were often noted on forms and kept separate to where the data was stored. All of the participants who had experienced reporting queries explained that this was to ensure that the queries never showed up in the database. For example, one participant confided:

“The desire for this kind of unwritten or unspoken ah, rule that if you had lots of queries you don’t want to an auditor to come in behind you and see all those queries” (P3).

### Working within regulatory requirements

#### Good clinical practice

Many participants described the GCP guideline as inflexible and the scope needed to be broadened as it mostly applied to drug trials. The participants felt completing GCP training was a dreary exercise. Despite this, they recognised that the guideline served a purpose and provided staff context to the overall structure of clinical studies. One participant voiced:

“There’s a general consensus and feeling that, the [GCP] guidelines are too strict. They have a purpose, but they are very much open to interpretation” (P3).

Participants also reflected on the importance of the GCP guideline in that it provides substantial trust to all procedures completed within a study. Participant responses illustrated the depth and stability of having a common set of guidelines:

“We often referred back to it [GCP]…to make sure we're doing this and people actually understood how that fitted in” (P4).

#### Protocol

Being able to create protocols to address study procedures was described as an easy process by one participant as they created the protocol from a template provided by the ethics committee. However, another participant explained that with experience they had begun to incorporate information specific to their area of research:

“That allowed us to adopt a whole range of more or less protocol defined approaches to all the activities relating to the design, implementation, conduct and reporting of clinical trials. So, the key thing I’d say is that that would reflect the differences is how naturally over that sort of 30 year period or 25 within the academic environment those [protocols] changed, or were modified in response to any many number of different stimuli.” (P2)

It was vital that studies implement protocols to ensure the study procedures are safe for the study subjects and data is of high quality. The majority of participants spoke about implementing and adhering to protocol defined approaches which were revised on a regular basis. Additionally, the significance of publishing the protocol was mentioned by the participants who had experienced this process and felt that the process ensured that the study design was clear and was followed.

“We want to put our processes and things on open science to ensure that we are very transparent about our protocols and procedures” (P5).

#### Standard operating procedure

SOPs were written in large organisations by senior and specialised staff members. One participant described that staff members who created SOPs often felt fatigue with the repetitive procedure. While another mentioned the irony that all SOPs and monitoring plans were similar. It was strongly argued that the SOP outlining the data monitoring plan was always a standalone document, providing clear instructions on how to carry out monitoring procedures.

“No, it [data monitoring plan] was always had a standalone SOP…around monitoring visits and frequency and so on, was a standalone document always.... And I’ve been in trials as I said since the mid 90s so that’s always been the case. I’ve never seen it any differently than that” (P3).

Participants reported that occasionally new staff members were resistant to introducing SOPs as they were naïve about their importance. The resistance was often reduced with training and education, resulting in participants calling for standardising documents and clearer guidance:

“I really think we need to be up to lift it up a bit [quality] and that I think if you can highlight that we need to have you know SOPs and standardise things [documents and procedures].” (P4).

Finally, SOPs were described as needing to be tailored to the study context, where the activities of the organisations were based on the resources available. When clinical studies were required to meet the same SOPs, staff felt resentment around meeting stricter requirements and demanding extra requirements. Not implementing context specific SOPs was reported as being problematic:

“I think the other difference of course is the way that the pharmaceutical industry with the benefit of substantial resources is able to operate is not at all how things can work in the academic environment. So, you have to create SOPs that people can actually work within and towards comfortably rather than try and emulate a pharmaceutical standard which would be inconceivably problematic in an academic environment” (P2).

## Discussion

From the interviews, we found that Australian organisations conducting intervention clinical trials which are testing new treatment options are implementing a variety of data quality monitoring procedures tailored to their clinical situation and study context. Participants experienced challenges in meeting regulatory requirements, utilising IT and fostering working relationships. Additionally, it was a common phenomenon for all clinical studies to lack guidance, education and training in relation to data quality monitoring procedures. Taken together, clinical researchers are calling for further education and training on data quality monitoring procedures.

Due to the unique and different needs of clinical studies, the participants described that data quality monitoring procedures were tailored to their clinical situation and study context. A “one-size-fits-all” approach to data quality monitoring was not applicable for all clinical studies. Moreover, participants expressed the need to meet regulations, particularly for large drug-intervention trials where a strict requirement is necessary to uphold and to meet the procedures outlined by the funding body and sponsorship agreements. Conversely, participants in smaller clinical studies described a more flexible setting where their studies were run subject to individual interpretation and allowed for incremental changes throughout study procedures. The present study, therefore, identifies a possible individual ‘enthusiasm factor’ related to the study researchers and coordinators that could positively impact on the quality of the data. In support of this notion a study in a primary care setting [38] identified that a chosen person who has the essential skills and eagerness to maintain data quality can lead and engage others to do so. This strategy has the potential to be used by small clinical studies where there is no designated data manager.

Participants voiced their challenges with meeting regulatory requirements and utilising technology to improve study data quality. Our participants experienced similar barriers that have been reported by previous researchers including meeting the demand for excessive monitoring, a lack of funding and inadequate infrastructure [39]. A lack of IT infrastructure has made it difficult for clinical studies to meet the required data monitoring procedures. This difficulty was keenly felt by the independent, non-commercial and academic small-scale researchers who work with limited budgets [40]. Such challenges may explain why no relationship was found in this study between the primary themes ‘working within regulatory requirements’ and ‘working with IT’ despite the GCP guidelines recommending a risk-based monitoring approach underpinned by an IT platform. Regardless of these challenges, participants expressed positive experiences with IT to improve data quality and to reduce error by improving transparency and building a level of trust between research communities and participants. Furthermore, the significance of having internationally recognised guidelines and procedures has meant that clinical staff understood the importance of project governance.

This study provides evidence for the positive impact that a good working relationship can have on data quality. Open communication between staff is crucial to the success of data monitoring. Additionally, the principal investigators working alongside other staff members was identified as a critical activity to promote successful study conduct and to maintain staff engagement. This result echoes findings that appropriate communication and advice promotes staff morale and enables collection of quality data [41, 42]. These lessons are useful for the contemporary clinical research study that is demanding increased need for collaboration.

Unfortunately, the participants experienced a lack of guidance, education and training. This result was not surprising as previous research has also reported a lack of understanding amongst clinical study researchers regarding the benefits of training on overall study performance [43]. Participants reported GCP training as tedious and not relevant. Additionally, some participants with experience of working within multidisciplinary environments reported that clinical staff may lack knowledge about research methods due to taking on research as additional work [44]. Little was found in the scientific literature about training and education for clinical study data quality monitoring. However, many companies do conduct GCP training course both online and in-person (e.g. PRAXIS [45], Quintiles [46], NIDA Clinical Trials Network [47] and ARCS Australia [48]). It is clear that an emphasis needs to be placed on available training courses which cater to clinical researcher’s different levels of expertise and roles in data collection and monitoring.

Our study had several limitations. Firstly, we had limited representation of clinical study types with all seven participants currently working on intervention treatment clinical trials. Therefore, the experiences of the participants may not be representative of the broader clinical research community, including the substantial number of intervention prevention, quality of life, screening, epidemiological, diagnostic and genetic clinical trials and observational studies. The participants were restricted to those who had previously completed the initial survey. The decision to not contact other professionals engaged in clinical research was made due to the design and linking between the two studies. This explanatory sequential research design provided the participants with the opportunity to expand and explain the context to their initial survey responses. Therefore, the small number of participants that were willing to be interviewed could have influenced the authors’ perceptions regarding thematic saturation. The use of small sample sizes and pragmatic participant recruitment in phenomenological research can allow for a rich and detailed exploration of individual experiences that do not aim to be representative or generalisable [49]. The findings of this study are subject to potential bias in a positive direction as those who were willing to participate in the interviews may have been more knowledgeable about data monitoring procedures and regulatory requirements than those who were not willing to participate. Secondly, this research was limited by participant bias as interviewees may have been hesitant to report negative experiences associated with their current or prior employer. The interviews were telephone-based, body language may have provided useful data which could not be assessed. Additionally, this was a retrospective study as participants were asked to reflect on their lived experiences. The retrospective design may be argued to be a limitation with regard to the trustworthiness of the findings [50]. As with any qualitative data the interviews and themes that emerged are subjective experiences of the interviewees and interviewer.

Together this article and the proceeding companion article have expanded on the information available about the current practices and barriers to data monitoring in Australian clinical research settings. Although both articles represent unique and significant contributions, they are a snapshot in time during a period of rapid advancement in national and international regulatory requirements and an expanding use of mobile and cloud-based information technologies. Further research in this field should explore barriers and facilitators for data quality monitoring in compliance with GCP regulation in different clinical settings and study contexts. Future research could be conducted to determine what is the most feasible, time and resource efficient education and training mechanism for clinical researchers to conduct data quality monitoring approaches.

## Conclusion

This study identified a variety of data quality monitoring procedures implemented by clinical researchers tailored to their clinical context. It also unveiled challenges experienced by clinical researchers in meeting regulatory requirements, utilising technology and fostering working relationships. At present, there is a lack of guidance for observational studies and non-drug intervention trials for data quality monitoring procedures. Standardised frameworks which are accessible to all clinical studies are warranted.

## Supplementary Information

**Additional file 1.** COREQ (COnsolidated criteria for REporting Qualitative research) Checklist.

**Additional file 2.** Online Semi-Structured Interview Guide.

**Additional file 3.** Interview Questions.

**Additional file 4.** Transcription Protocol.

**Additional file 5.** Hierarchical structure of the primary themes, secondary themes and subthemes.

## Data Availability

The datasets used and/or analysed during the current study are available from the corresponding author on reasonable request.

## Abbreviations

ANZCTR: Australian and New Zealand Clinical Trial Registry
COREQ: COnsolidated criteria for REporting Qualitative research guidelines and checklist
CRF: Case report form
GCP: Good Clinical Practice
ICH: International Council for Harmonisation
IT: Information technology
SDV: Source data verification
SOP: Standard Operating Procedure
