# Supplementary material for: Clinical researchers’ lived experiences with data quality monitoring in clinical trials: a qualitative study
Source: BMC Med Res Methodol. 2021 Sep 20;21:187. doi: 10.1186/s12874-021-01385-9 (PMC8454069; doi:10.1186/s12874-021-01385-9)
Supplement: Supplementary file 2 — Additional file 2. Online Semi-Structured Interview Guide. [file 12874_2021_1385_MOESM2_ESM.pdf]

UNIVERSITY
OF WOLLONGONG
AUSTRALIA

Additional file 2

Online Semi-Structured Interview Guide

Introductory Statement

Hi [name],

Firstly, thank you very much for taking the time and agreeing to take part in this study. As you probably know, my name is Lauren and I am currently completing the second year of my PhD at the University of Wollongong, which this research is part of.

I understand that you have already read and signed the Participant Information Sheet and Participant Consent Form. Did you have any questions about any of the information mentioned in either of those? [pause]

I will just read you an overview of the plans for today's interview so you know what to expect. Just to clarify the purpose of this research is to understand how clinical researchers experience data quality monitoring in Australia. In particular, today's interview questions will focus on auditing tools and procedures, which include staff training. We will also touch on the motivators and barriers to ensure quality assurance of clinical research data.

During our interview, I will be asking you about your experience with the monitoring of data quality before, during and after a clinical study. I will ask you to describe your experiences that have been successful and/or unsuccessful. I will then finish up talking to you about any education and training that you may have undertaken related to data quality.

The findings from this research will be used as part of my PhD thesis, and will be published in scientific journals and presented at suitable conferences. Ultimately, my research aims to develop a toolkit for clinical researchers to improve data quality monitoring in Australia.

I also just wanted to remind you that we are focusing on your experience in monitoring data quality in clinical studies, so this includes both intervention trails and observation studies. I am not assessing you knowledge of what is right or wrong I am purely trying to work out what is happening in the clinical research space. In addition, to make sure that the privacy of any people or organisations you are and or have worked with is protected I will remove these from the interview before analysis.

Before we start, based on what I have just read do you have any questions? [pause] Can I confirm that you still agree to participate in this interview? [pause]

I also want to remind you that your participation today is voluntary and that you may stop the

interview at any time without any consequence. If you would like to stop the interview, or feel like you need to take a break, please just let me know. Similarly, if there is a question that you do not feel comfortable answering please let me know too. Finally, if there are any technical difficulties and something does go wrong, please tell me by sending me an email and hopefully we can fix it for you.

I expect our interview today to take between 30 and 60 minutes. Is that okay with you? [pause] I will check with you after 30 minutes to make sure you are okay for time.

Can I also check with you that you are happy to have this interview recorded today? [pause]

[If participant does not agree to recording] Can I check if you are happy about me writing notes from today’s interview by hand and sending them by email to you for review? [pause]

[If participant agrees to recording] The recording helps to make sure I can transcribe what was said in our interview today. The recording will be stored securely at the University of Wollongong and all of the data from this recording, including your interview transcript, will be de-identified. We will be using your participant code to analyse the data to ensure your privacy. The file that maps your code to your name will also be kept securely and separately from the rest of the data. My supervisors and I will be the only people who have access to the data, and your name will not appear in any transcripts, my thesis, publications or presentations.

[Recording on] Thank you for agreeing to have this interview recorded, I will start recording now [turn on recording devices if participant agrees].

[Recording off] Thank you for agreeing to me handwriting notes from this interview, I will start taking notes now.

Do you have any questions you would like to ask before we start? [pause]

Interview Questions

[Name] to start with today I’ll be asking you some introductory questions. Firstly, it would be great to know about the journey you have had working in clinical research.

Probes:

- ☐ How you came to working in clinical research
- ☐ Drove interest in working in this area
- ☐ Range of clinical research settings / physical environment
- ☐ Range of clinical research type (intervention/observation)
    - Intervention - type (diagnostic, epidemiological, genetic, prevention, quality of life, screening, treatment)

As I mentioned earlier, I am particularly interested in understanding your experience with monitoring data quality. For the purpose of today's interview, I am going to refer to 'data quality monitoring' as the oversight and review of research processes, procedures, records, data reporting, appropriate conduct and ongoing evaluation. How do you feel about this definition? [pause]

So now, can you think about the clinical research setting/range of settings [adapted to suit] you just mentioned that you have work in and could you comment broadly on your individual experience with monitoring data quality.

Probes:

- ☐ Similarities and differences
- ☐ Aspects of your work specifically data quality

So now, the following questions follow on from the answers you provided in the online survey earlier this year. Please keep in mind that the survey answers were linked to a particular clinical study in which yours was [trial name]. The difference today is that this interview aims to understand more in depth your personal experiences and points of view.

<u>Data definition</u>

From your survey answers, you have told me that you implement/don't implement/follow/don't follow [adapt to individual survey answer]. It would be great if you could describe your experience with such data quality procedures that are implemented before a clinical study starts.

Probes:

- ☐ Reflect on how this experience happened/role in this process
- ☐ Reflect on the setting and context
- ☐ Reflect on successful and not so successful
- ☐ An example of an experience which worked well or didn't work

<u>Data collection</u>

I would now like to move onto data collection, which as you know is the process of accumulating data elements. Your survey answers show that the clinical study has [adapt to individual survey answer]. Could you describe your experience with such procedures over your research career?

Probes:

- ☐ An example for overcome missing values during data collection
- ☐ Reflect on technology (paper, mobile, electronic)
- ☐ Reflect on data management tools (cost, generic, built own)

Data processing

In your survey answers, you mentioned that the clinical study implements [adapt to individual survey answer] to audit and monitor data. Could you describe your involvement with this/these method(s)?

Probes:

- ☐ Reason why [name method is chosen and completed
- ☐ Reason for implementing more than one method
- ☐ Experience with other methods/reason for change
- ☐ Key factors of method

Data representation

Now I would like to focus on your experience with data analysis and translating data into information for dissemination. Your survey answers indicate that you [adapt to individual survey answer] could you comment on your role and your opinion about such procedures.

Probes:

- ☐ Reflect on a definition for ‘poor data quality’
- ☐ Reflect on an error acceptance level
- ☐ Reflect on continuous quality improvement
- ☐ Reason why selection of the three most important factors that impact data quality answer

Education and training

Given what we have spoken about today and hearing about your experiences, I’d like you to reflect on any training you have received regarding data entry, data quality or data monitoring. Can you tell me about these experience(s)?

Probes:

- ☐ What the experience involved
- ☐ Usefulness of the training
- ☐ Areas of training (skills, procedures, SOPs, ICH GCP)
- ☐ Reflect on training delivery (group, one-on-one)

Closing Statement

That takes us to the end of our interview. Thank you very much [name] for agreeing to be interviewed today. You have given me some great insights into your experience with monitoring data quality as a clinical researcher. Before we finish up, can I ask if there were anything else, you would like to add. [pause]

If you have any further questions or think of anything you would like to add please do not hesitate to contact me by email after the interview.

Before you go, I need to remind you that the recording from today's interview will be transcribed. If you would like to review your transcript, I can email it to you so you can confirm that you are happy with what has been transcribed.

Thanks very much again [name], enjoy the rest of your day.
