# Supplementary material for: Clinical researchers’ lived experiences with data quality monitoring in clinical trials: a qualitative study
Source: BMC Med Res Methodol. 2021 Sep 20;21:187. doi: 10.1186/s12874-021-01385-9 (PMC8454069; doi:10.1186/s12874-021-01385-9)
Supplement: Supplementary file 3 — Additional file 3. Interview Questions. [file 12874_2021_1385_MOESM3_ESM.pdf]

# Additional file 3

# Interview Questions

This document outlines the questions you will be asked during your telephone interview by the interviewer, Lauren Houston. You have been provided these questions before your scheduled telephone interview to help you in understanding what is involved when completing the interview, and to give you some time to reflect and develop your responses if you wish, given these questions ask about your specific experiences before, during and after a clinical study.

Please do not hesitate to contact Lauren Houston on [telephone number] or by [email address] if you have any questions.

### Data quality monitoring definition

The oversight and review of research processes, procedures, records, data reporting, appropriate conduct and ongoing evaluation.

### Interview questions

1. Could you tell me about your journey you have had working in clinical research?
2. Could you comment broadly on your individual experience with monitoring data quality?
3. Could you describe to me your experience with data quality procedures that are implemented before a clinical study starts?
4. Could you describe your experience with data quality procedures during the data collection phase?
5. Could you describe to me your involvement with any methods implemented to audit and monitor data?
6. Could you comment on your role and opinion with any data quality procedures you have been involved in during data analysis phase and translating data into information for dissemination?
7. Could you reflect on any training you have received regarding data entry, data quality or data monitoring. Can you tell me about these experience(s)?
