# Supplementary material for: Clinical researchers’ lived experiences with data quality monitoring in clinical trials: a qualitative study
Source: BMC Med Res Methodol. 2021 Sep 20;21:187. doi: 10.1186/s12874-021-01385-9 (PMC8454069; doi:10.1186/s12874-021-01385-9)
Supplement: Supplementary file 4 — Additional file 4. Transcription Protocol. [file 12874_2021_1385_MOESM4_ESM.pdf]

Additional file 4

Transcription Protocol

This protocol follows the outline of the Sample Qualitative Data Preparation and Transcription Protocol published in 'Beyond the Qualitative Interview: Data Preparation and Transcription' by McLellan, MacQueen and Neidig (2003).

Ms Lauren Houston shall transcribe all interview recordings which will be made from an audio recording.

1. Text formatting

General Instructions

The following format in Microsoft Word will be adhered to by the transcriber for all interviews:

Calibri (Body) font, size 12

Margins: Top and bottom – 2.5cm, left – 2.5cm, right – 6.0cm

All text shall begin at the left hand side margin (no indents)

All text shall be left justified

Line numbers (excluding coversheet)

Page numbers in footer and right justified (excluding coversheet)

Coversheet of interview transcripts

The interview transcript shall include a coversheet as the first page of the document, and include the following information:

The transcript title 'Interview Transcript' in bold text, size 18

Participant identification code

Date and time of interview

Location of interview

Interviewer name

Transcriber name

Reviewer name

Medium, all interviews conducted via telephone

Number of interviews with participant

If transcript approved by the participant (if requested)

Date the transcript was originally transcribed

Date transcript was checked

Date transcript was reviewed

Field notes

Key thoughts

Example of transcript coversheet:

Interview transcript

Participant code: ID-0000

Date and time of interview: 1st January 2018, 12:00pm

Location: 41.309 UoW Science Building

Interviewer name: Lauren Houston

Transcriber name: Lauren Houston

Reviewer name: John Smith

Medium: Telephone interview

Number of interviews with participant: 1/1

Transcript approved by participant: Not requested

Date transcribed: 01st September 2018

Date transcript checked by transcriber: 14th October 2018

Date transcript checked by reviewer: 10th November 2018

Field notes:

XXX

Key thoughts:

XXX

Start of Transcript

The transcriber shall indicate the beginning of the interview recording and transcript, by typing the words '*START OF INTERVIEW RECORDING'* at the start of a new page immediately after the coversheet. The text will be written in uppercase, italic, size 16 and centre justified.

End of Transcript

When the interview has reached completion, the transcriber shall indicate by typing '*END OF RECORDED INTERVIEW'* on the last line immediately after the last spoken text. The text will be written in uppercase, italic, size 16 and centre justified.

Source Labelling

The individual participant identification code shall indicated by 'ID-' followed by the appropriate four digit code provided in the prior survey to the participant, for example ID-0000. The code will be written in uppercase and immediately before the spoken text on the same line followed by a colon.

The interviewer shall be indicated by the code 'INTERVIEWER'. The code will be written in upper case and immediately before the spoken text on the same line followed by a colon.

2. Content

Interview recordings will be transcribed verbatim, to include every recorded word, exactly as said and nonverbal or background sounds. This includes the following:

Verbal Content

All filler words such as 'um', 'ah', 'oh', 'mm', 'mhmm', 'yeah', 'huh' will be transcribed.

Mispronounced words will be transcribed as pronounced. If mispronunciation results in difficulties with understanding of the text, the correct word shall be typed immediately after in square brackets. *E.g. I thought that was pretty pacific [specific], but they disagree.*

Any foul language, slang and/or swear words shall be written as said and will not be "cleaned up".

Unfinished or cut off words will be transcribed and a hyphen shall be inserted at the end of the last letter or audible sound. *E.g. he wen- he went and did what I told him to do).*

Repeated words or phrases shall be transcribed. *E.g. so ah for example, I think that, that you shouldn't…*

Obvious shortening of words and informal contractions will be transcribed as said. *E.g. 'gonna' meaning going to.*

Non-Verbal Content

No non-verbal or background sounds will be transcribed in square brackets. *E.g. [laugh], [sigh], [cough].*

Inaudible Information

Speech that is difficult to be recognised or deciphered as an explicit word or short phrase by the transcriber shall indicate as “inaudible” at the point of speech where it occurred in square brackets. *E.g. what I call a [inaudible] shop is where I go shopping.*

Overlapping Speech

If there is simultaneous speaking or overlapping of speech recorded and the transcriber cannot hear clearly or distinguish what each person is saying the transcriber shall indicate as “cross talk” at the point of speech where it occurred in square brackets. *E.g. [cross talk].*

Pauses

Short pauses between speaking shall be indicated by a comma.

Longer pauses between 2-5 seconds of speaking shall be indicated at the point of speech where it occurred through the use of three ellipse. *E.g. Such as then . . . um, we would*

Substantial delays shall be transcribed with action or “delay” at the point of speech where it occurred in square bracket. *E.g. [toilet break] or [delay].*

Technical issues/Freezing

If there is a technical issue, prolonged unexplained silence (greater than 10 seconds) or freeze in the recording it shall be indicated as a “freeze” at the point of speech where it occurred in square brackets. *E.g. [freeze].*

Sensitive information

If names of people, locations of work or any other identifiable information are spoken of in the discussion, the transcriber shall replace to ensure confidentiality at the point of speech where it occurred in square brackets. *E.g. [name], [location].*

3. Transcript reviewing (quality assurance and control)

Three stages of transcript reviewing:

1. The transcriber will check and proof read transcripts against the audio recording for accuracy. Any revisions that are required will be made to ensure the transcript reflects the audio recording.

2. A reviewer will then check the transcription against the audio recording for accuracy. The second reviewer will use tracked changes in Microsoft Word to document any recommended

changes. The transcriber and reviewer will discuss any discrepancies identified before transcript is amended.

3. If requested by the participant, the transcript and audio recording will be sent to the participant for checking. Any discrepancies or amendments requested by the participant will be made in tracked changes to the document and returned to the primary researcher.

A Microsoft Excel spreadsheet will be kept to document all three stages for the transcript reviewing process. Documentation will include dates the transcript was checked at each stage and notes regarding recommended changes and amendments.

4. Saving the transcripts

All transcripts will be saved in a Microsoft Word file. The final copy of the transcript approved by the transcriber and reviewer, and if requested by the participant will be saved in both a Microsoft Word file and a Portable Document Format (PDF).

All transcript files will be named with the participant code, initials of those who have checked the transcript, and the day, month and year of checked transcript (DDMMYY). The final version of the transcript will be indicated with the name FINAL.
E.g.

Unchecked transcript: Transcript ID-0000

Stage 1: Transcript ID-0000 LH 141018

Stage 2: Transcript ID-0000 LH 141018 YP 01022019

Final: Transcript ID-0000 FINAL
