# Supplementary material for: Clinical researchers’ lived experiences with data quality monitoring in clinical trials: a qualitative study
Source: BMC Med Res Methodol. 2021 Sep 20;21:187. doi: 10.1186/s12874-021-01385-9 (PMC8454069; doi:10.1186/s12874-021-01385-9)

Additional file 5

Hierarchical structure of the primary themes, secondary themes and subthemes

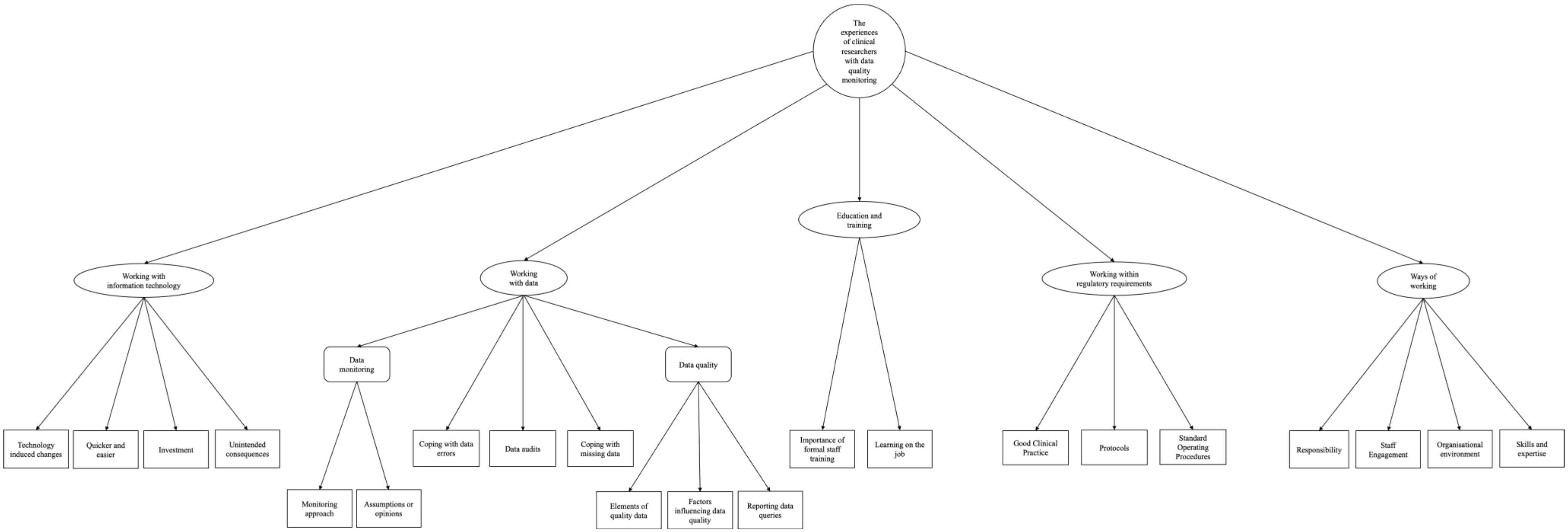

Supplement: Supplementary file 5 — Additional file 5. Hierarchical structure of the primary themes, secondary themes and subthemes. [file 12874_2021_1385_MOESM5_ESM.pdf]
